# Enhanced anti-tumor immune responses and delay of tumor development in human epidermal growth factor receptor 2 mice immunized with an immunostimulatory peptide in poly(D,L-lactic-co-glycolic) acid nanoparticles

**DOI:** 10.1186/s13058-015-0552-9

**Published:** 2015-03-31

**Authors:** Diahnn F Campbell, Rebecca Saenz, Ila S Bharati, Daniel Seible, Liangfang Zhang, Sadik Esener, Bradley Messmer, Marie Larsson, Davorka Messmer

**Affiliations:** Moores UCSD Cancer Center, University of California San Diego, 3855 Health Sciences Drive, La Jolla, CA 92093-0815 USA; Department of Nanoengineering, University of California San Diego, 3855 Health Sciences Drive, La Jolla, CA 92093-0815 USA; Department of Bioengineering, University of California San Diego, 3855 Health Sciences Drive, La Jolla, CA 92093-0815 USA; Division of Molecular Virology, Department of Clinical and Experimental Medicine, Sandbäcksgatan 7, Linköping University, 581 83 Linköping, Sweden; Inception Sciences, 5871 Oberlin Drive, San Diego, CA 92121 USA

## Abstract

**Introduction:**

Cancer vaccines have the potential to induce curative anti-tumor immune responses and better adjuvants may improve vaccine efficacy. We have previously shown that Hp91, a peptide derived from the B box domain in high-mobility group box protein 1 (HMGB1), acts as a potent immune adjuvant.

**Method:**

In this study, Hp91 was tested as part of a therapeutic vaccine against human epidermal growth factor receptor 2 (HER2)-positive breast cancer.

**Results:**

Free peptide did not significantly augment immune responses but, when delivered in poly(D,L-lactic-co-glycolic) acid nanoparticles (PLGA-NPs), robust activation of dendritic cells (DCs) and increased activation of HER2-specific T cells was observed *in vitro*. Vaccination of HER2/*neu* transgenic mice, a mouse breast cancer model that closely mimics the immune modulation and tolerance in some breast cancer patients, with Hp91-loaded PLGA-NPs enhanced the activation of HER2-specific cytotoxic T lymphocyte (CTL) responses, delayed tumor development, and prolonged survival.

**Conclusions:**

Taken together these findings demonstrate that the delivery of the immunostimulatory peptide Hp91 inside PLGA-NPs enhances the potency of the peptide and efficacy of a breast cancer vaccine.

**Electronic supplementary material:**

The online version of this article (doi:10.1186/s13058-015-0552-9) contains supplementary material, which is available to authorized users.

## Introduction

Vaccines are a promising approach to prevent or cure cancer [1,2] but generally require a tumor antigen and an immune-stimulatory adjuvant. Breast cancers that express the human epidermal growth factor receptor 2 (HER2) have been treated with some success by immunotherapies that target that antigen [3]. Vaccines can be potentiated by their method of administration and formulation. For example, nanoparticles (NPs) can protect sensitive/and or unstable antigens such as peptides from degradation and potentially increase the immune response to vaccines. It has been shown that encapsulation of antigen into biodegradable spheres leads to enhanced humoral and cellular immune responses [4,5]. Poly(D,L-lactic-co-glycolic) acid nanoparticles (PLGA-NPs) have been used to deliver the cancer-associated antigen MUC1,5,6 as well as tetanus toxoid to enhance immune responses [6]. PLGA is a biodegradable and biocompatible polymer [7,8] with good stability in the gastrointestinal tract [9] and is used for numerous *in vivo* applications [10,11]. NPs also have the advantage that, by using different polymer compositions, one can control the release of cargo allowing for antigen depot formation at the injection site. These manipulations might provide enabling technologies to the vaccine as well as drug development field.

Dendritic cells (DCs) are the most potent antigen-presenting cells and are critical for the initiation of adaptive immune responses. Vaccines need to stimulate DCs to induce potent immune responses. DCs must receive a maturation signal to present antigen, upregulate costimulatory and adhesion molecules, and become potent activators of T cells [12]. The immunostimulatory peptide Hp91, which is derived from the endogenous protein high-mobility group box protein 1 (HMGB1), activates DCs [13] and primes antigen-specific cytotoxic T lymphocyte (CTL) responses *in vitro* [13] and *in vivo* [14]. Hp91 packaged inside of PLGA-NPs is more potent in activating DCs as compared to free peptide [15]. In our previous study, the PLGA-NPs were synthesized using an emulsion method yielding non-homogeneous particles. In the current study, we used a precipitation method that yields homogeneous NPs, to package Hp91 inside PLGA-NPs. We evaluated the extent to which Hp91-PLGA-NPs protect against breast cancer using a HER2 breast cancer mouse model [16]. Our results demonstrate that the delivery of the immunostimulatory peptide Hp91 inside the PLGA-NPs enhances the efficacy of this breast cancer vaccine.

## Materials and methods

### Peptides

The adjuvant peptide Hp91 (DPNAPKRPPSAFFLFCSE) and MHC class I (H2-D^q^)-restricted rat HER-2/*neu*-derived peptide (PDSLRDLSVF) were both purchased from CPC Scientific (San Jose, CA, USA). The Hp91 peptide was synthesized with an N-terminal biotin and dissolved in RPMI for *in vitro* studies and phosphate-buffered saline (PBS) for immunizations. The HER2 peptide was dissolved in 3% dimethyl sulfoxide (DMSO)/PBS. Peptides were routinely synthesized with greater than 95% purity.

### Animals

FVB.N/*neu*-tg mice were derived from in-house breeding stocks at the University of California, San Diego (UCSD) Moores Cancer Center animal facility. All animal studies were approved by the Institutional Animal Care and Use Committee of the University of California, San Diego and performed in accordance with the institutional guidelines.

### Synthesis of peptide-loaded lipid-polymer hybrid nanoparticles

Ester-terminated poly-lactic-co-glycolic acid, or PLGA (50:50, 0.82 dl/g IV, DURECT Corporation, Cupertino, CA, USA) was dissolved at 1 mg/ml in dimethylformamide (DMF). Hp91 was also dissolved in DMF with the PLGA at concentrations of 1 to 5 mg/ml. Lecithin (molecular weight (MW) 330 Da, Alfa Aesar, Ward Hill, MA, USA) and 1,2-distearoyl-sn-glycero-3-phosphoethanolamine-N-(carboxy(polyethylene glycol)2000) (ammonium salt) (DSPE-PEG-Carboxy, MW 2,849.54 Da, Avanti Polar Lipids, Alabaster, AL, USA) were dissolved together in 2 ml of 4% ethanol per mg PLGA to be used at a ratio of 9% of total PLGA weight for lecithin and 52% of total PLGA weight for DSPE-PEG-Carboxy. All stock solutions were made using sterile solvents or endotoxin-free water. The aqueous lipid mixture was heated to 68°C while stirring for 3 min. The PLGA-peptide solution was added dropwise to the heated lipid solution while stirring. The solution was then vortexed at 3,000 RPM for three minutes. An additional 1 ml of water per mg of PLGA used was added dropwise to the NP solution while stirring. The NP solution was stirred without cap for 2 h to allow solvent evaporation. The particles were then washed three times using Amicon Ultra centrifugal filter devices by EMD Millipore (Billerica, MA, USA) with 100 Kd cutoff. Particles were suspended in 10% sucrose and flash frozen for later use.

### Characterization of lipid-polymer polylactic-co-glycolic acid hybrid nanoparticles (PLGA-NPs)

The NP formation was analyzed for particle size by dynamic light scattering (DLS) using a zetasizer (Zetasizer Nano ZS, Malvern Instruments Ltd, Malvern, UK). To quantify the amount of peptide loaded into the hybrid NPs, the NPs were dissolved in DMF for 30 min under constant shaking at room temperature and peptide content was quantified by high-performance liquid chromatography (HPLC) (column: Waters Delta-Pak C18 5 microns, Waters Corporation, Milford, MA, USA) at 211 nm in comparison to a Hp91 peptide standard curve. To measure the release rate of the peptide from the NPs, 100 μL of Hp91-loaded NP solution was added to microdialysis cassettes with a MW cutoff of 10,000 and dialyzed against 1 L of PBS buffer at pH 7.4 or potassium hydrogen phthalate buffer at pH 5. At each time point, two samples for each buffer condition were recovered from the microdialysis cassettes, and the volumes were brought up to 125 μL to keep all volumes constant. To each sample, 125 μL of DMF was added to dissolve the NPs and release the remaining Hp91 peptide. The samples were shaken for 60 min, and then the total amount of Hp91 in each sample was quantified using HPLC. The amounts were normalized against the starting concentration of peptide before dialysis, which was set at 100% to calculate the percentage released.

### Generation of mouse bone marrow-derived DCs

Bone marrow-derived dendritic cells (BM-DCs) were prepared from HER-2/*neu* transgenic mice (H-2^q^), as described by Inaba *et al*. [17] with minor modifications. Briefly, single bone marrow cell suspensions were obtained from femurs and tibias, depleted of lymphocytes, granulocytes, and Ia + cells using a mixture of monoclonal antibodies (mAbs; anti-CD4, anti-CD8, anti-B220/CD45R, and anti-Ia) for 45 min on ice, followed by incubation with low-toxicity rabbit complement for 30 min at 37°C. Cells were resuspended at a concentration of 10^6^ cells/mL in medium supplemented with recombinant murine granulocyte-macrophage colony-stimulating factor (GM-CSF) (10 ng/mL). Fresh medium (5% vol/vol fetal calf serum (FBS)-RPMI) containing GM-CSF was added on day 2 and 4 of culture. On day 6, cells were collected for the experiments.

### Antigen presentation assays

Immature BM-DCs (10^5^) were stimulated with media alone, similar amounts of Hp91 free peptide, NP-encapsulated Hp91, or 10 ng/mL lipopolysaccharide (LPS) (Sigma-Aldrich, St Louis, MO, USA). Forty-eight hours after activation, the cells were incubated with 100 ng/mL HER2 peptide for 1 h at 37°C. The cells were then washed twice to remove excess peptide and plated with HER2-specific CTL clones (kindly provided to us by Professor E. Jaffee (John Hopkins Medical Institute) at a 10^3^:10^4^ DC to T cell ratio in wells of a nitrocellulose bottom enzyme-linked immunospot (ELISPOT) plate (EMD Millipore) that had been previously coated overnight with 5 μg/mL monoclonal anti-mouse interferon gamma (IFN-γ) antibody (Mabtech, Stockholm, Sweden). After 18 h, the ELISPOT plates were developed using 1 μg/ml biotinylated anti-mouse IFN-γ antibody (Mabtech), Streptavidin-horseradish peroxidase (HRP) (Mabtech), and 3,3',5,5'-Tetramethylbenzidine (TMB) substrate (Mabtech). The plate was scanned and the spots were counted using an automated ELISpot Reader System (CTL ImmunoSpot, Shaker Heights, OH, USA).

### Immunizations and spleen cell preparation

The HER-2/*neu* peptide antigen was co-administered subcutaneously with either PBS, soluble Hp91, or NP-encapsulated Hp91 on the right flank. Spleens were collected 8 days after the final immunization. Single cell suspensions of splenocytes were prepared by mechanical disruption and separation through a 70-μm nylon cell strainer (BD Biosciences, Franklin Lakes, NJ, USA). Red blood cells were lysed using ammonium chloride buffer (Roche Diagnostics, Indianapolis, IN, USA) and the splenocytes were subsequently resuspended in complete medium (RPMI 1640 with 10% FBS, L-glutamine, penicillin, streptomycin, and HEPES) supplemented with 20 U/mL of recombinant mouse interleukin (IL)-2) (R&D Systems, Minneapolis, MN, USA) and 10 μg/mL of PDSLRDLSVF peptide for expansion. Splenocytes were expanded for 5 days prior to use in ELISPOT experiments.

### Enzyme-linked immunospot assay

The expanded splenocytes were collected and washed twice before being plated in duplicate 10^6^ cells to wells of an ELISPOT plate that had been previously coated overnight with 5 μg/mL monoclonal anti-mouse IFN-γ antibody. Splenocytes were cultured overnight at 37°C with 2.5 μg/mL HER2 peptide, 5 μg/ml concavalin A (Sigma-Aldrich) as positive control or left unstimulated (medium only). After 18 h, ELISPOT plates were developed using 1 μg/ml biotinylated anti-mouse IFN-γ antibody, streptavidin-HRP, and TMB substrate. The plate was scanned and the spots were counted using an automated ELISpot Reader System.

### Tumor prevention experiments

Female HER-2/*neu* mice, 8 weeks of age, were immunized with 5 μg of HER2 antigen mixed with either PBS only, 25 μg of Hp91 free peptide, or 25 μg of Hp91 delivered in PLGA-NPs. Mice received their first boost 2 weeks post-prime, and a second boost 1 month thereafter. All injections were performed subcutaneously on the right flank of the mice. The incidence and growth of tumors were evaluated twice a week by measuring palpable tumors, defined as tumors with diameters that exceed 3 mm, with calipers in two perpendicular diameters. Calipers were used to measure tumor length and width and the volume was calculated as volume (mm^3^) = (width)^2^ × length/2. All mice bearing tumor masses exceeding 1.5 cm mean diameter were sacrificed.

### Statistical analysis

Data represented are mean ± standard error of the mean (SEM). Data were analyzed for statistical significance using unpaired Student’s *t* test. Statistical analyses were done using GraphPad software version 5.01 for Windows (GraphPad Software, San Diego, CA, USA). A *P* value <0.05 was considered statistically significant for these analyses.

## Results

### Delivery of Hp91 in PLGA-NPs increases its immunostimulatory capacity *in vitro*

**Figure 1:**
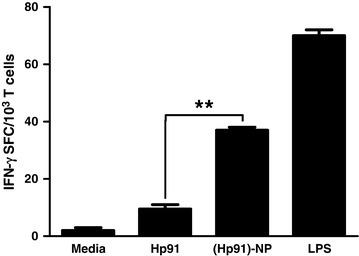
Delivery of Hp91 in nanoparticles to BM-DCs increases HER2-specific CTL responses *in vitro.* Immature mouse BM-DCs (10^5^) were exposed to media, 55 μg/ml of free Hp91 peptide or the same amount delivered inside PLGA-NPs, or lipopolysaccharide (LPS; 10 ng/mL). After 2 days the cells were collected, washed, and pulsed with HER2 peptide (100 ng/mL) for 1 h prior to the overnight co-culture with the HER-2/*neu*-specific CTL line in an ELISPOT assay. The number of IFN-γ spot-forming cells is shown as means (± standard error of the mean (SEM)) of two independent experiments. ^**^Indicates statistical significance *P* <0.005. BM-DCs, bone marrow-derived dendritic cells; CTL, cytotoxic T lymphocyte; ELISPOT, enzyme-linked immunospot; HER2, human epidermal growth factor receptor 2; IFN-γ, interferon gamma; PLGA-NPs, poly(D,L-lactic-co-glycolic) acid nanoparticles.

We have previously shown that delivery of the immunostimulatory peptide Hp91 in PLGA-NPs leads to increased activation of both mouse and human DCs *in vitro* [14]. Those NPs were made using an emulsion method, which results in NPs that are very heterogeneous in size [15]. To develop a vaccine that can be taken into the clinic, it is critical to synthesize homogenous particles. Therefore, we have evaluated a nanoprecipitation method to incorporate Hp91. This method generates very homogeneous PLGA-NPs (Figure S1B in Additional file 1). Hp91 was efficiently released at pH 5 and faster release was observed at pH 7.4 (Figure S1A in Additional file 1). To examine whether Hp91 maintains its DC-stimulatory properties [14,18] when packaged inside NPs, immature human monocyte-derived DCs were exposed to increasing doses of either free Hp91 peptide or Hp91-loaded NPs. Hp91 incorporated in PLGA-NPs caused increased activation of DCs *in vitro* as measured by increased secretion of IL-6, as compared to free peptide (Figure S1C in Additional file 1). Next, we investigated whether the encapsulation of Hp91 could enhance HER2-specific T cell responses. BM-DCs from HER2/*neu* transgenic mice were assessed for their ability to induce antigen-specific T cell responses as measured by IFN-γ secretion in an ELISPOT assay. DCs were exposed to media, free Hp91 peptide or encapsulated in PLGA-NPs or LPS for 48 h. The DCs were then washed and pulsed with HER-2/*neu* peptide and tested for their ability to activate antigen-specific CTL responses using a HER-2/*neu*-specific CTL line. Hp91 loaded inside PLGA-NPs was significantly more potent in activating DCs, generating nearly a 4-fold increase in HER-2/*neu-*specific CTL responses when compared to Hp91 delivered in its free form (Figure 1, *P* <0.005).

### Hp91 delivered in PLGA-NPs increases immune responses to the HER2/*neu* antigen *in vivo*

**Figure 2:**
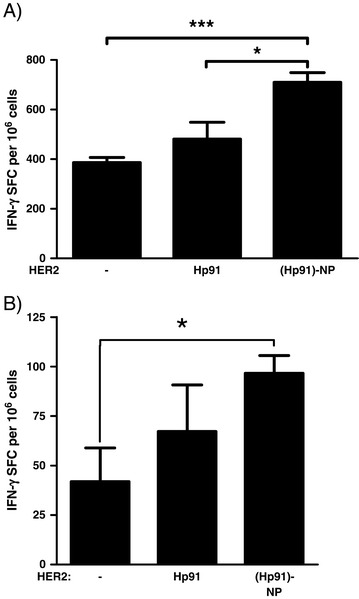
Immunization of HER2/neu transgenic mice with Hp91 in nanoparticles increases CTL responses. **(A)** HER2/*neu* transgenic mice at 2 months of age were immunized with 5 μg HER2 peptide in PBS, or with 25 μg Hp91 free peptide or the same amount of Hp91 packaged within PLGA-NPs. **(B)** A different group of HER2/*neu* transgenic mice was immunized at 4 months of age with 20 μg HER2 free peptide or 4.5 μg HER2 peptide inside PLGA-NP in PBS, alone or in combination with 250 μg Hp91 free peptide or 14 μg Hp91 in PLGA-NPs. **(A** and **B)** Eight days after the final immunizations, mice were sacrificed and expanded splenocytes were cultured in the presence of HER2 peptide in an IFN-γ ELISPOT assay. CTL responses were determined 18 h later by quantifying the number of IFN-γ spot-forming cells using an automated ELISPOT reader. Data shown represents the means (± standard error of the mean (SEM)) for five mice per group. ^*^Indicates statistical significance *P* <0.05. CTL, cytotoxic T lymphocyte; ELISPOT, enzyme-linked immunospot; HER2, human epidermal growth factor receptor 2; IFN-γ, interferon gamma; PBS, phosphate-buffered saline; PLGA-NPs, poly(D,L-lactic-co-glycolic) acid nanoparticles.

The transgenic HER2/*neu* mouse model was chosen as it had been shown to closely mimic the immune modulation and tolerance described in some breast cancer patients [16]. We tested if Hp91 when delivered in PLGA-NPs would be sufficiently potent to break tolerance and induce potent CTL responses *in vivo*. HER2/*neu* transgenic mice were vaccinated at three different time points starting at 2 months of age with HER2 peptide mixed with free Hp91, Hp91-loaded PLGA-NPs, or PBS control. Splenocytes from immunized mice were analyzed for HER2-specific CD8+ T cell responses by IFN-γ ELISPOT assay (Figure 2A). Although an increase in immune response was observed when free Hp91 was co-injected with the HER2 peptide antigen, it was not statistically significant. However, when mice were injected with a similar dose of Hp91 encapsulated inside PLGA-NPs together with HER2 peptide antigen, a significant increase in HER2-specific IFN-γ spot-forming cells as compared to the control group was measured (*P* <0.0001) (Figure 2A). In addition, encapsulation of Hp91 peptide inside PLGA-NPs also enhanced the immune response when compared to mice immunized with free Hp91 (*P* = 0.015) (Figure 2A). Next, we tested whether the free Hp91 peptide at higher doses could induce immune responses in the HER2 mice. The mice were immunized according to the schedule above, but with a 10-fold higher dose of 250 μg of free Hp91 and 14 μg of Hp91 packaged in PLGA-NPs (Figure 2B). Even at higher doses free Hp91 peptide did not induce significant CTL responses in this mouse model. However, when packaged inside of PLGA-NPs even at an 18-fold lower dose of Hp91 as compared to free peptide, induced a significant increase in CTL responses. This shows that when delivered in PLGA-NPs the immunostimulatory peptide Hp91 shows increased adjuvant potency *in vivo*.

### Immunization of HER2/*neu* mice with Hp91 loaded in PLGA-NPs delays tumor development and prolongs survival

**Figure 3:**
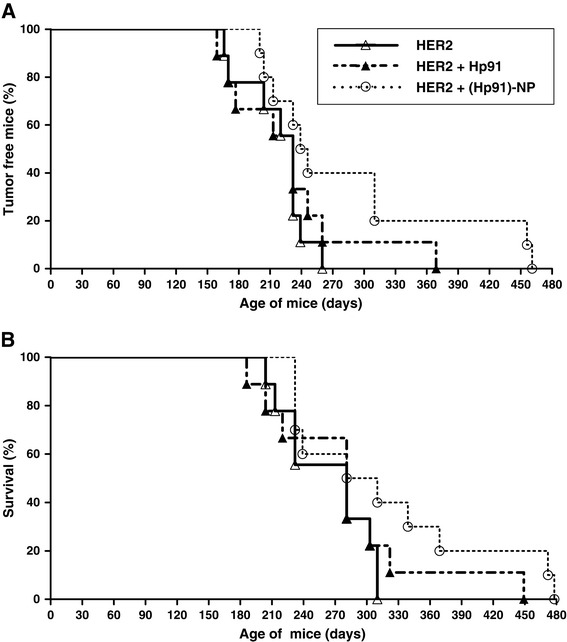
Immunization of HER2/neu-transgenic mice with Hp91 in nanoparticles delayed tumor development and prolonged survival. At 2 months of age, mice were immunized with 5 μg HER2 peptide in PBS, 25 μg of Hp91 free peptide, or 25 μg Hp91 delivered in PLGA-NPs. After final immunization, mice were monitored twice a week for tumor growth and survival. **(A)** Percentages of tumor-free mice were calculated as cumulative number of mice with tumor and mice that were tumor free. **(B)** Mice were sacrificed once tumor masses exceeded 1.5 cm mean diameter. HER2, human epidermal growth factor receptor 2; PBS, phosphate-buffered saline; PLGA-NPs, poly(D,L-lactic-co-glycolic) acid nanoparticles.

The transgenic HER2/*neu* mice spontaneously develop mammary tumors beginning at 4 months of age. Therefore, we sought to investigate if our nanoparticle vaccine could prevent or delay tumor development in these mice. Mice received subcutaneous (s.c.) injections of the different vaccine compositions at 2 months of age and were monitored for development of palpable tumors twice a week. Mice were sacrificed once tumor masses exceeded 1.5 cm mean diameter. By 8.5 months of age, all mice from the control HER2 peptide antigen only group had developed tumor masses. Although all mice vaccinated with free Hp91 together with free HER2 peptide developed tumors, the vaccine delayed tumor development by approximately 2 months in some mice in comparison to mice immunized with the HER2 peptide antigen only control group (Figure 3A). Immunization of mice with Hp91 packaged in PLGA-NPs mixed with free HER2 peptide delayed the measurable appearance of tumors by an additional approximately 3 months compared to control mice and prolonged the overall survival (Figure 3B). These results indicate that the immunostimulatory peptide Hp91 when packaged inside of PLGA-NPs significantly enhances the anti-tumor response to free HER2 peptide in a prophylactic vaccine setting and yielded the longest survival (Figure 3B). Mice immunized with just the HER2 antigen alone were all sacrificed by approximately 10 months of age due to tumor mass. Increased immune responses correlated with protection from tumor development correlated.

## Discussion

The activation of potent immune responses by vaccines or therapies is essential and not always achieved by traditional vaccines or therapies. The use of NPs to deliver adjuvant and antigen is now being thoroughly investigated. Tumor-associated antigens have been identified for a variety of cancers but so far very few clinical trials have shown great efficacy. This could have many reasons including immune suppression by the tumor microenvironment, poorly immunogenic antigens, limited T cell repertoire and so on. Another possibility could be deficiency in the vaccine delivery systems. The immune system generally responds to viruses and bacteria, which are particles and not isolated molecules and they contain the antigen in close proximity to the adjuvant. Various NPs are being explored as delivery tools for drugs, vaccine antigens and adjuvants. NPs are a delivery platform that can be tuned for different needs. The material used to make NPs can in itself be immunogenic, for instance poly(γ-glutamic acid) activates the immune system via TLR4/MyD88 NP [19]. Our goal was to use inert materials like PLGA-NPs as a neutral carrier, which can be loaded with immunogenic moieties, in this case the Hp91 peptide, to trigger desired immune responses. One reason for using non-stimulatory materials and loading them with adjuvant is that we believe understanding the mechanism behind material-induced immune responses is difficult, and using characterized adjuvants should be of advantage. The PLGA-NPs used in this study are biodegradable and biocompatible polymer10-13 that has been employed for numerous *in vivo* applications. We have not observed immune activation with the PLGA-NPs alone [16]. The PLGA-NPs used were 100 nm (range 30 to 200 nm), a size which has been shown to be preferentially taken up by professional antigen-presenting cells (APCs) and are expected to enter DCs or Langerhans cells (LCs) when given transcutaneous or s.c..

We found that Hp91, when packaged inside of PLGA-NPs, activated both mouse and human DCs *in vitro* and induced increased CD8+ T cell responses as compared to free peptide. One possible explanation is that the delivery is more efficient, because the NPs are readily taken up by DCs and each NP will deliver many peptides, whereas free peptide will diffuse around the cells and the uptake is much less effective. Interestingly, the intensity of CTL responses seen in the immunized mice mirrored protection from tumor development.

## Conclusions

Vaccination with HER2 peptide combined with Hp91-loaded PLGA-NPs broke tolerance against the HER2 antigen in the HER2/*neu* transgenic mice and prolonged survival. This vaccine warrants further investigation for efficacy in other models as well as for safety. If proven safe in clinical trials, an interesting consideration would be to vaccinate patients with precancerous lesions as, for example, patients with HER2-positive ductal carcinoma *in situ*. These patients will likely have a fully functional immune system and should show good response to the vaccine whereas late-stage HER2-positive patients like any late-stage cancer patients will have an impaired immune responses and lower likelihood of success. Combinations with new therapies like anti-PD1 or anti-CTLA4 antibodies might yield better success in late-stage patients.

## Additional file

Additional file 1: Figure S1. Characterization of PLGA-NPs. **(A)** Release of Hp91 peptide from PLGA-NPs and **(B)** PLGA-NP size was measured as previously described [18]. **(C)** Immature DCs were exposed to PLGA-NP-loaded Hp91 or the equivalent amount of free peptide for 48 h. Cell culture supernatants were analyzed for IL-6 by ELISA.

## Abbreviations

APCs: antigen-presenting cells
BM-DCs: bone marrow-derived dendritic cells
CTL: cytotoxic T lymphocyte
DCs: dendritic cells
DLS: dynamic light scattering
DMF: dimethylformamide
DMSO: dimethyl sulfoxide
DSPE-PEG-Carboxy: 1,2-distearoyl-sn-glycero-3-phosphoethanolamine-N-(carboxy(polyethylene glycol)2000)
ELISPOT: enzyme-linked immunospot
FBS: fetal bovine serum
GM-CSF: granulocyte-macrophage colony-stimulating factor
HER2: human epidermal growth factor receptor 2
HMGB1: high-mobility group box protein 1
HPLC: high-performance liquid chromatography
HRP: horseradish peroxidase
IFNγ: interferon gamma
IL: interleukin
LCs: Langerhans cells
LPS: lipopolysaccharide
mAbs: monoclonal antibodies
MW: molecular weight
NP: nanoparticle
PBS: phosphate-buffered saline
PLGA: poly(D,L-lactic-co-glycolic) acid
PLGA-NPs: poly(D,L-lactic-co-glycolic) acid nanoparticles
s.c.: subcutaneous
TMB: 3,3',5,5'-Tetramethylbenzidine
