# Supplementary figures and images for: Enhanced anti-tumor immune responses and delay of tumor development in human epidermal growth factor receptor 2 mice immunized with an immunostimulatory peptide in poly(D,L-lactic-co-glycolic) acid nanoparticles
Source: Breast Cancer Res. 2015 Mar 31;17(1):48. doi: 10.1186/s13058-015-0552-9 (PMC4407876; doi:10.1186/s13058-015-0552-9)

## Slide 1
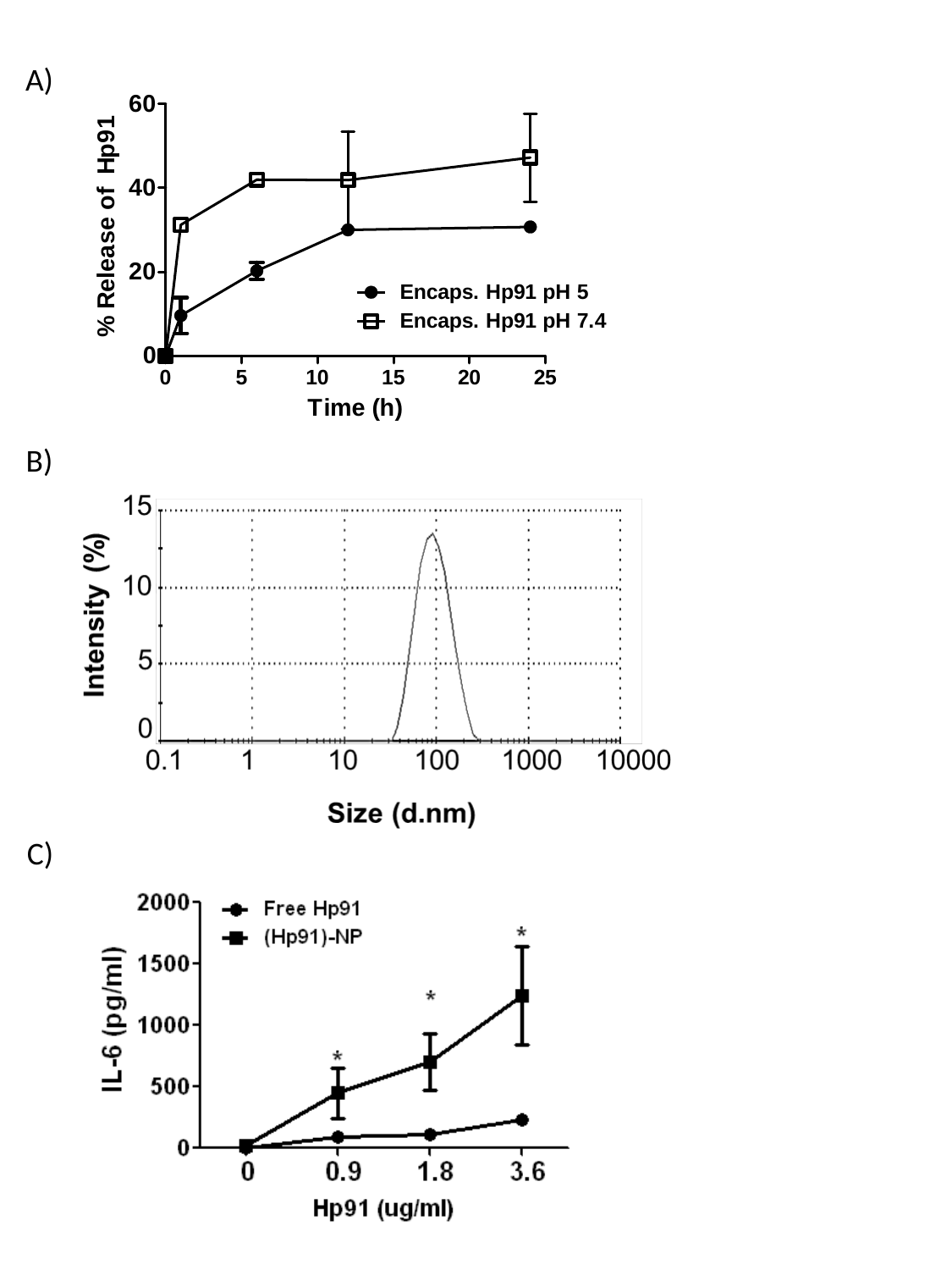

A)
B)
C)

Supplement: Additional file 1: Figure S1. — Characterization of PLGA-NPs. (A) Release of Hp91 peptide from PLGA-NPs and (B) PLGA-NP size was measured as previously described [18]. (C) Immature DCs were exposed to PLGA-NP-loaded Hp91 or the equivalent amount of free peptide for 48 h. Cell culture supernatants were analyzed for IL-6 by ELISA. [file 13058_2015_552_MOESM1_ESM.pptx]
